# Research Progress on Target Gene Screening and the Precise Control Potential of *Diaphorina citri* Using RNA Interference

**DOI:** 10.3390/insects17090953

**Published:** 2026-09-12

**Authors:** Liping Ye, Zhenrui He, Xiang Li, Aijun Huang, Jun Zhou, Long Yi

**Affiliations:** 1 College of Life Sciences, Gannan Normal University, Ganzhou 341000, China; 18989310284@163.com (L.Y.); x18317513414@163.com (X.L.); huangaijun@gnnu.edu.cn (A.H.); 13102188352@163.com (J.Z.); 2 Institute of Plant Protection, Jiangxi Academy of Agricultural Sciences, Nanchang 330200, China; zhenruihe@163.com

**Keywords:** *Diaphorina citri*, Huanglongbing, RNA interference, dsRNA, sustainable pest management

## Abstract

*Diaphorina citri* Kuwayama is the primary natural vector of citrus Huanglongbing (HLB). Prolonged use of chemical pesticides leads to insect resistance, reduces control efficacy, and causes ecological pollution. RNA interference (RNAi) enables sequence-specific gene silencing, representing a promising precision strategy for managing *D. citri*. This review summarizes advances in RNAi-based control of *D. citri*, focusing on three groups of target gene screening strategies based on essential functional genes, behavioral regulation genes, and *D. citri*-*Candidatus* Liberibacter asiaticus (*C*Las) interaction genes. It also compares double-stranded RNA (dsRNA) delivery systems and highlights key bottlenecks, including poor stability, inefficient delivery, off-target effects, and resistance evolution. Future research should focus on optimizing target gene screening, developing efficient delivery systems, and enhancing ecological safety assessments. Integrating RNAi technology with various environmentally friendly management strategies will advance the sustainable control of *D. citri* and citrus HLB.

## Simple Summary

Citrus Huanglongbing (HLB) is a devastating disease threatening global citrus production. The pathogen is mainly transmitted by *Diaphorina citri* Kuwayama. Chemical insecticides are widely used to suppress this psyllid, yet long-term application drives insecticide resistance, reduces control efficacy and causes ecological pollution. This review focuses on an environmentally friendly and precise biocontrol strategy based on RNA interference. We summarize feasible target gene screening approaches, analyze major technical constraints hindering field deployment, and outline prospects for further improvements, to facilitate sustainable management of citrus pests and diseases.

## 1. Introduction

Citrus Huanglongbing (HLB), commonly known as citrus greening disease, is the most destructive and incurable bacterial disease worldwide, severely hindering the sustainable development of the global citrus industry [1,2,3]. HLB causes severe tree decline, fruit malformation, and substantial yield loss, often leading to complete orchard failure in severely affected citrus-growing regions and resulting in enormous economic losses [4,5,6]. Under natural field conditions, *Diaphorina citri* Kuwayama (Asian citrus psyllid) serves as the primary natural vector of HLB [7,8]. The feeding, dispersal, and reproduction behaviors of *D. citri* are primarily responsible for the widespread transmission and epidemic outbreak of HLB [9,10]. Therefore, effective suppression of *D. citri* populations and interruption of pathogen transmission are critical for the integrated management of citrus HLB.

Currently, chemical pesticide application remains the predominant strategy for controlling *D. citri* and limiting HLB spread due to its rapid efficacy and convenient field operation [11,12]. However, long-term and intensive reliance on synthetic pesticides imposes persistent selective pressure on *D. citri* populations, which accelerates the evolution of insecticide resistance and gradually reduces field control efficacy [8,13]. To maintain pest management efficiency, increased pesticide dosages and frequent applications are commonly adopted in practice, which in turn exacerbates agroecological pollution, reduces farmland biodiversity, elevates pesticide residue risks, and threatens food safety and ecological balance [14]. The inherent limitations of conventional chemical control have rendered it incompatible with the green and sustainable development goals of modern citrus production, highlighting an urgent demand for innovative, efficient, and environmentally friendly alternative strategies to improve current *D. citri* and HLB management systems.

RNA interference (RNAi) is a sequence-specific gene silencing mechanism that precisely suppresses the expression of target genes, thereby interfering with insect growth, development, reproduction, and behavioral traits to achieve targeted pest control [15,16,17,18]. Featuring high species specificity, operational flexibility, and ecological safety, RNAi has emerged as a promising precision biocontrol technology to overcome the drawbacks of traditional pest management [19,20]. RNAi-based biopesticides are widely regarded as the “third pesticide revolution”, driving a sustainable approach for modern crop protection [21]. Representative commercial products, such as SmartStax PRO-the first commercially approved RNAi-enabled corn targeting western corn rootworm-and registered formulations like DP23211 corn and VT4PRO, have demonstrated the feasibility and field efficacy of sequence-specific RNAi in pest management. In recent years, extensive studies have advanced RNAi-based strategies for *D. citri* control, and numerous functional target genes have been identified and validated for *D. citri* suppression [22,23]. Nevertheless, the practical application of RNAi technology still faces multiple bottlenecks, including poor dsRNA stability, insufficient delivery efficiency, potential off-target effects, and the risk of resistance evolution in *D. citri* [24,25,26,27]. Furthermore, unsystematic target gene screening frameworks and imperfect ecological safety evaluation systems greatly restrict the field popularization and large-scale implementation of RNAi-based HLB and psyllid management.

This review systematically summarizes recent research progress on RNAi-based technologies for *D. citri* control. We elaborate three core target gene screening strategies based on essential functional genes, behavioral regulatory genes, and genes involved in the *D. citri*-*Candidatus* Liberibacter asiaticus (*C*Las) interaction. In addition, we comprehensively compare the advantages and limitations of diverse dsRNA delivery systems and systematically highlight major application bottlenecks restricting field promotion. Future research priorities are proposed to optimize target gene screening systems, develop high-efficiency delivery platforms, and improve ecological safety assessment protocols. Integrating RNAi technology with diversified green pest management strategies will provide new insights for the sustainable and precise control of *D. citri* and citrus HLB.

## 2. Target Gene Screening for RNAi-Mediated *Diaphorina citri* Control

### 2.1. Screening Strategies and Technical Methods of Target Genes

Target gene screening constitutes the primary and core step for the establishment of efficient RNAi-based pest control systems [28,29]. Nevertheless, the screening and application of *D. citri* target genes still face multiple inherent limitations. Notably, RNAi efficiency exhibits significant spatiotemporal heterogeneity, as psyllids display distinct RNAi sensitivity across different developmental stages and diverse tissue types, which substantially affects the stability and repeatability of gene silencing effects [30,31]. In addition, non-specific gene silencing caused by off-target effects frequently distorts the functional verification results of candidate genes, thereby reducing the accuracy of target gene screening. The detection of potential off-target risks generally relies on high-throughput sequencing and multi-omics verification, which necessarily increases the experimental cost and technical difficulty of large-scale target gene screening [32,33].

**Figure 1:**
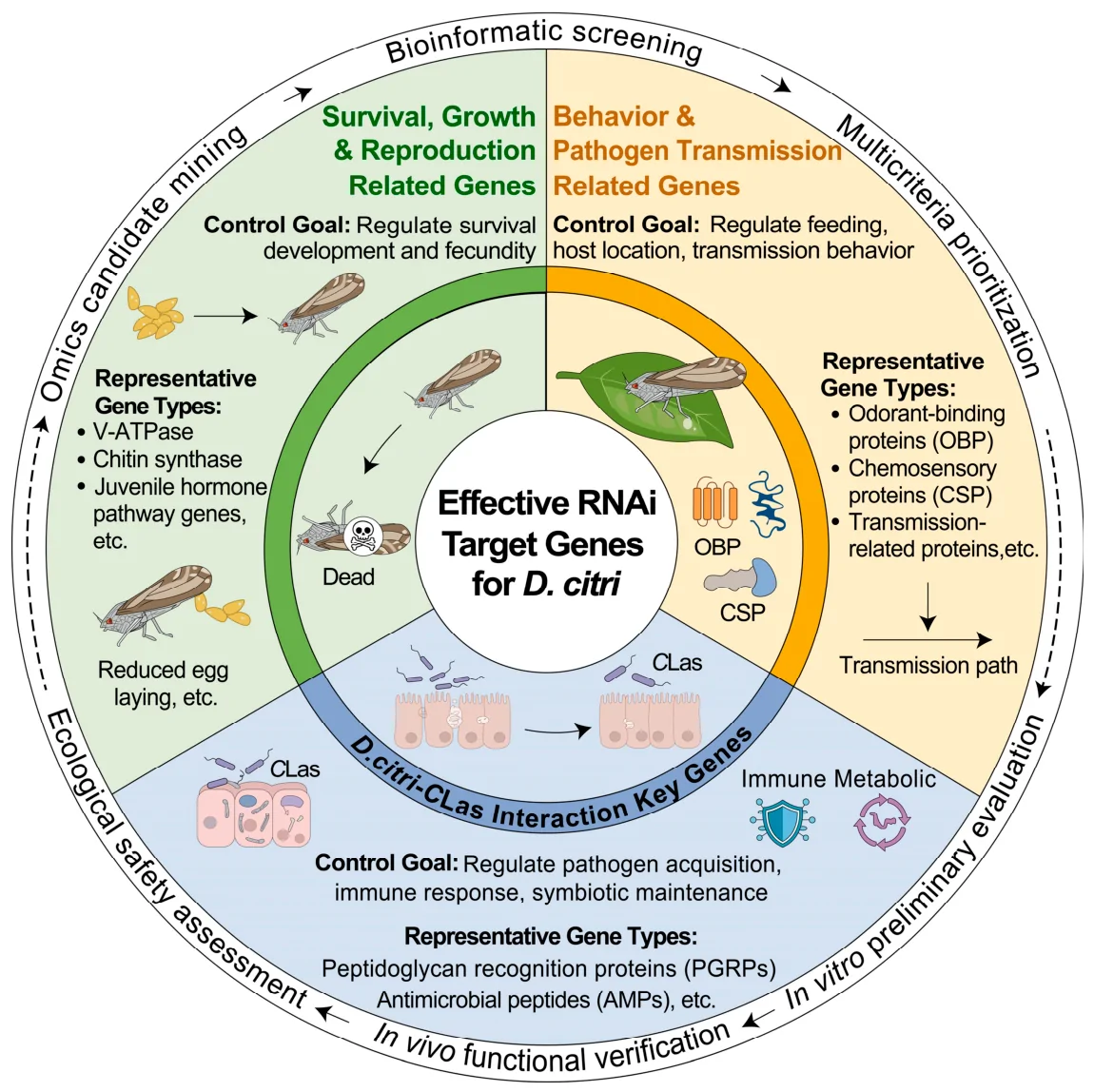
Three functional categories of effective RNAi target genes in *Diaphorina citri*. The validated target genes are classified into three modules: genes controlling survival, growth and reproduction of *D. citri* (green sector), genes modulating psyllid behaviors and *Candidatus* Liberibacter asiaticus (*C*Las) transmission (orange sector), and genes mediating the interaction between *D. citri* and *C*Las (blue sector).

Currently, the screening of RNAi target genes for *D. citri* follows a stepwise strategy from broad preliminary mining to precise functional validation [30]. First, candidate gene libraries are constructed based on the biological characteristics of *D. citri* and its interactive relationship with *C*Las. Genomic and transcriptomic datasets are systematically excavated to mine potential target genes that regulate psyllid survival, development, behavior, and pathogen transmission. Subsequently, bioinformatic prediction tools and in vitro cell models are adopted for high-throughput primary screening of massive candidate genes. Candidate genes are further prioritized according to multiple evaluation criteria, including lethal efficiency, suppression of feeding and behavioral performance, and the capacity to block *C*Las transmission. For high-priority candidate genes, in vivo functional verification is conducted on *D. citri* nymphs and adults. Specifically, dsRNA is chemically synthesized and delivered via oral feeding or microinjection to assess gene silencing efficiency and corresponding phenotypic alterations. Finally, the optimal target genes are screened out, and their ecological safety toward non-target organisms and host citrus plants is comprehensively evaluated, ensuring the efficacy, biosafety, and environmental compatibility of RNAi application [34,35,36,37]. Collectively, this technical system forms a complete screening workflow covering omics-based target mining, bioinformatic prioritization, in vitro and in vivo functional verification, and ecological safety assessment (Figure 1).

After sequential screening via the above multi-step screening and functional validation system, the experimentally validated candidate target genes of *D. citri* are mainly categorized into three major groups: genes essential for insect survival, growth, and reproduction; key genes regulating psyllid behavioral traits and pathogen transmission; and pivotal genes involved in the *D. citri*-*C*Las interaction (Figure 1). The functional characteristics of these three types of target genes will be elaborated in detail in the following Section 2.2. Bioinformatic prediction and sequence analysis serve as the fundamental basis for the entire screening process. With the available whole-genome data of *D. citri*, homologous sequence alignment is commonly performed to mine candidate genes based on functionally validated target genes from other insect species. Before experiments, virtual screening is implemented to narrow down candidate genes efficiently at a low cost [29,38]. During this process, professional bioinformatic software is used to evaluate the sequence specificity of designed dsRNA fragments, eliminating sequences with high homology to non-target organisms to minimize off-target risks. Meanwhile, the secondary structure stability of dsRNA sequences is predicted to avoid structural constraints that may hinder binding with core RNAi machinery and compromise gene silencing efficiency [39,40,41,42]. The species specificity of RNAi primarily originates from the strict sequence complementarity required between dsRNA derived siRNAs and their target mRNA molecules. Off-target silencing occurs when short sequence fragments of siRNA partially match non-target gene transcripts. Therefore, bioinformatic screening during the target gene selection stage serves as the primary preventive measure: dsRNA fragments with high sequence homology to genes of non-target arthropods, beneficial insects, and citrus host plants should be excluded in advance to minimize the theoretical risk of cross-species off-target effects.

### 2.2. Categories and Control Efficacy of Validated Key Target Genes

Precise screening and functional verification of target genes constitute the core of RNAi-mediated pest management. Identifying the classification and insecticidal efficacy of high-efficiency target genes serves as an essential prerequisite for constructing efficient *D. citri* control systems and blocking the spread of citrus HLB. The selection and efficacy evaluation of target genes directly determine RNA silencing efficiency and final pest control outcomes, and those functionally validated high-potential target genes can provide theoretical support for the development of RNAi pesticides and the optimization of field application schemes in subsequent research.

**Figure 2:**
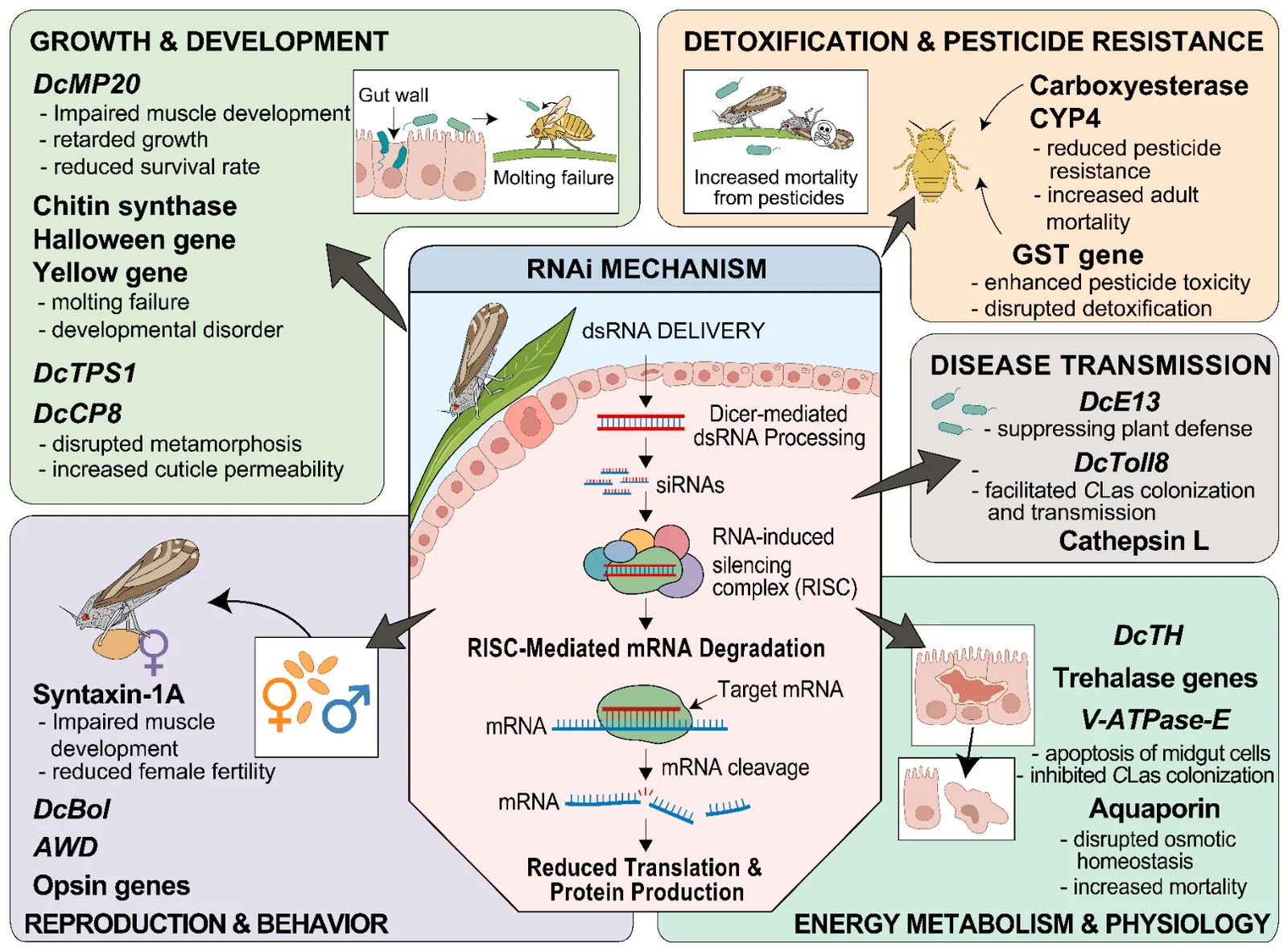
The mechanism of RNA interference and functional classification of verified target genes in *Diaphorina citri*. Ingested dsRNA is processed into siRNAs to activate the RNA-induced silencing complex and degrade target transcripts. Validated RNAi target genes are classified into five major functional groups. Gene silencing induces multiple detrimental phenotypes in *D. citri* and inhibits the transmission of *Candidatus* Liberibacter asiaticus.

At present, verified RNAi target genes of *D. citri* cover multiple physiological pathways, ranging from growth and development, energy metabolism and reproduction to behavioral perception, detoxification metabolism and immune interaction with *C*Las (Figure 2). Collectively, these validated targets are grouped into the three major functional categories summarized above, and can be further subdivided into five functional sub-categories. Among growth and development-related genes, silencing the muscle protein 20 gene (*DcMP20*) via dsRNA soaking suppresses the normal muscle development of psyllid nymphs. This repression reduces nymph body weight and survival rate, maintains persistent gene downregulation after treatment, disrupts myoblast fusion during muscle formation, and ultimately leads to delayed development and death of *D. citri* [43]. As for chitin synthase gene, silencing *DcCHS* inhibits chitin biosynthesis of citrus psyllid, triggers cuticle ultrastructural damage and molting abnormality, elevates nymph mortality, and provides a feasible target for RNAi-mediated prevention and control against *D. citri* [44]. Ecdysone synthesis-related *Halloween* gene and pigment gene *yellow* are high-efficiency lethal targets; their silencing hinders molting, triggers developmental disorder and dehydration, resulting in mass death of nymphs and adults [45,46]. Silencing the trehalose-6-phosphate synthase gene (*DcTPS1*) causes molting abnormality and malformed phenotypes in nymphs, increased mortality, together with disturbed chitin and fatty acid metabolism of psyllids [47,48]. Silencing the RR-2 subfamily cuticular protein gene *DcCP8* inhibits exocuticle synthesis, disrupts adult metamorphosis, increases cuticle permeability, and suppresses chitin metabolism-related gene expression, while star polycation nanoparticles-wrapped dsRNA-mediated *DcCP8* silencing elevates *D. citri* mortality by activating endocytic and immune pathways [49]. Apart from genes associated with growth and development, multiple target genes related to reproduction and behavioral perception have also been characterized in *D. citri*. The vesicle-fusion-associated protein Syx1A, a functional reproductive regulator of *D. citri*, can impair yolk protein deposition and ovarian maturation, suppress female fecundity, generate high mortality across nymph and adult stages, and greatly reduce population reproduction capacity of *D. citri* [30]. The reproduction-related gene *DcBol*, a conserved meiotic regulator in *D. citri*, mediates spermatogenesis; however, its silencing increases adult mortality without significantly impairing male fecundity [50]. The wing development-related abnormal wing disk (*awd*) gene mediates flight capacity; its RNAi silencing triggers adult wing malformation and impairs the flight performance of *D. citri* [51]. Meanwhile, opsin genes are critical for psyllid phototactic behavior, and dsRNA-mediated knockdown of these genes significantly reduces phototactic response efficiency [52]. Numerous detoxification-related genes participate in xenobiotic metabolism and mediate pesticide resistance in *D. citri*. RNAi targeting multiple *CYP4* genes represses cytochrome P450 protein abundance and oxidase activity, attenuating insecticide resistance and increasing adult mortality [53]. Topical delivery of dsRNA targeting esterases FE4 (*EstFE4*) and acetylcholinesterases (*AChe*) specifically downregulates the respective gene transcripts and induces concentration-dependent nymphal mortality [54]. In addition, the glutathione S-transferase genes *DcGSTd1* and *DcGSTe2* are responsible for detoxifying fenpropathrin and thiamethoxam, and simultaneous silencing of these two genes further improves the insecticide susceptibility of psyllids [55].

Verified RNAi target genes in *D. citri* also include genes implicated in disease transmission. The salivary effector DcE13, a functional salivary protein of *D. citri*, can suppress the jasmonic acid defense pathway of citrus, weaken plant anti-insect resistance, build favorable conditions for *C*Las infection, and greatly elevate the transmission efficiency of HLB [56]. Recent studies have demonstrated that the Toll signaling pathway is indispensable for the immune defense against *C*Las infection. Knockdown of the expression of *DcToll8* facilitates *C*Las colonization and substantially promotes pathogen transmission by psyllids [22]. Correspondingly, a functional cathepsin L in *D. citri*, acts as a crucial immune effector that directly cleaves the *C*Las outer membrane protein BamD, contributing to the host’s innate defense against bacterial infection and restricting pathogen proliferation in psyllids [23]. Furthermore, silencing the *DcCHC* gene impairs endocytosis and endosome network functions in *D. citri*, suppresses *C*Las survival and proliferation in psyllid midgut and whole body, and effectively reduces the *C*Las titer during pathogen acquisition [57]. Genes regulating energy metabolism and fundamental physiological processes have also been validated as effective RNAi targets in *D. citri*. Knockdown of the metabolism-related *V-ATPase-E* gene induces apoptosis of midgut epithelial cells and disrupts nutrient absorption, resulting in weight loss, physiological exhaustion and death of *D. citri*, while simultaneously inhibiting the systemic colonization of *C*Las inside psyllids [58]. Trehalase genes belonging to carbohydrate and energy metabolism genes are vital targets for lethal RNAi intervention. Silencing trehalase genes *DcTre1-1*, *DcTre1-2* and *DcTre2* disturbs trehalose homeostasis, suppresses chitin metabolism, and induces developmental malformations in *D. citri* [59]. Silencing the aquaporin gene *DcAQP* disrupts osmotic homeostasis, elevates nymph mortality, induces adult malformation and shortens adult lifespan in *D. citri* [42]. In addition, silencing tyrosine hydroxylase gene *DcTH* suppresses exocuticle synthesis and cuticle tanning, impairs metamorphosis, and increases mortality, making it a promising RNAi target for *D. citri* [60].

Overall, the currently identified RNAi target genes of *D. citri* encompass multiple key physiological and biochemical pathways closely associated with individual growth, population reproduction, behavioral adaptation, pesticide resistance, and pathogen transmission. Functional silencing of these genes via RNAi technology can effectively induce developmental defects, physiological dysfunction, and increased mortality in *D. citri*, while also impairing the vector’s ability to transmit *C*Las. Notably, distinct target genes exhibit significant differences in insecticidal efficiency and functional specificity, with a small number of genes showing unsatisfactory control effects. This provides a comprehensive theoretical reference for the screening, verification, and prioritized application of high-efficiency and specific RNAi target genes. Furthermore, these findings establish a solid foundation for the development of novel, environmentally friendly pest control technologies targeting *D. citri* and the sustainable management of citrus HLB disease [29].

From the perspective of translational application, target-gene prioritization should be established based on defined pest-control objectives. Two major application orientations can be distinguished: population suppression through direct lethality and HLB epidemic management via pathogen-transmission interruption. For target gene evaluation, comprehensive indicators should be considered, including RNAi silencing efficiency, phenotypic intensity, effective action stages of psyllids, sequence specificity, and potential off-target risks. When the primary goal is rapid reduction in field psyllid populations, priority should be given to targets that produce strong lethal phenotypes. By contrast, if the core objective is to block *C*Las dispersal and contain HLB spread, more attention should be paid to genes participating in pathogen acquisition, colonization and transmission. Balancing these evaluation criteria helps to screen out candidate genes with high practical transformation value for subsequent dsRNA formulation development and field testing.

## 3. Delivery Systems of RNAi Technology for *D. citri* Control

### 3.1. dsRNA Preparation, Laboratory Delivery Methods and Efficacy Verification

Following the identification of candidate target genes, dsRNA synthesis and delivery become two pivotal procedures for RNAi implementation. In vitro transcription serves as the dominant and reliable strategy to produce dsRNA at present. Briefly, target gene fragments fused with the T7 promoter are amplified by PCR using *D*. *citri* cDNA as the template, and high-purity dsRNA is subsequently synthesized in large quantities with commercial in vitro transcription kits [61,62]. Successful intracellular delivery of dsRNA is essential to activate RNAi responses in laboratory trials. Restrictions still exist for existing delivery techniques, and improving RNAi efficacy mainly relies on optimizing delivery pathways and protecting dsRNA against nuclease degradation [63]. Moreover, RNA silencing efficiency varies distinctly among different genes. Multiple intrinsic properties of target transcripts, including mRNA secondary structure, mRNA half-life, expression abundance and subcellular localization, jointly determine the final silencing effect [64,65,66].

Five classic laboratory delivery techniques are widely adopted: oral feeding, microinjection, soaking, nanocarrier-mediated delivery and plant-mediated RNAi. The oral feeding method mixes dsRNA into artificial diets for ingestion by psyllids. It causes no physical injury to insects and mimics natural exposure conditions, yet its performance is constrained by feeding behavior variability and intestinal degradation of dsRNA [67]. Microinjection delivers quantified dsRNA solution directly into the hemocoel or specific tissues for systemic distribution, which works for nymphs and adults of all developmental stages. Nevertheless, this technique suffers high technical requirements, inevitably damages psyllids, consumes tremendous labor, and cannot be applied to large-scale treatment. Nanocarrier delivery is an emerging technique developed to stabilize dsRNA and boost delivery efficiency. Forming dsRNA-nanomaterial complexes enables dsRNA protection from environmental nucleases and facilitates transmembrane penetration across the intestinal epithelium or body wall of psyllids via feeding or spraying [49]. The soaking method immerses psyllid eggs or young nymphs in dsRNA solution, allowing dsRNA uptake through eggshells and cuticles driven by osmotic pressure [68]. Plant-mediated RNAi generates psyllid-targeted dsRNA inside host citrus plants via stable transformation or transient expression systems; this approach closely simulates field application scenarios but requires lengthy cultivation cycles [69]. Exogenous sprays on plant foliage and water solutions applied to plant roots for viable field-relevant RNAi treatments have also been reported [70,71,72].

The efficacy of RNAi needs systematic verification across molecular and phenotypic layers. Upon dsRNA treatment, quantitative real-time PCR is performed to quantify the transcript abundance of target genes and determine gene silencing efficiency [73]. However, diminished mRNA abundance does not always translate to lowered protein production, which necessitates validation at the protein level. Western blotting is adopted to detect the decline of target proteins, and immunofluorescence staining serves as a semi-quantitative alternative when specific antibodies cannot be obtained [74,75]. Particularly for screening targets intended to halt HLB spread, it is necessary to further detect the capacity of dsRNA-treated psyllids to acquire and transmit *C*Las, which acts as a critical indicator for evaluating the practical value of candidate target genes. Collectively, laboratory preparation and delivery systems establish a complete system for RNAi functional verification. Different delivery approaches each possess distinct advantages and limitations, and multi-index comprehensive identification ensures objective evaluation of gene silencing effects. Relevant laboratory findings build a solid theoretical foundation for the optimization and popularization of field delivery modes in the next stage. Although the laboratory delivery methods described above are effective under controlled conditions, their translation to field environments faces multiple obstacles.

### 3.2. Exploration and Optimization of Field Delivery Systems

Excellent silencing phenotypes obtained under controlled laboratory conditions often fail to be reproduced in field environments, which constitutes a major bottleneck restricting the practical application of RNAi against *D. citri*. Microinjection, a mature laboratory delivery method, cannot be scaled for orchard application due to the tiny body size and active mobility of psyllids. Complex field conditions including rainfall leaching, ultraviolet radiation and fluctuating pH values readily degrade naked dsRNA and impair RNAi efficacy. By contrast, soaking delivery is labor-saving and suitable for high-throughput gene screening at the laboratory scale, but it cannot be directly transferred to orchard field conditions. This laboratory-based method has been verified for managing multiple agricultural pests in experimental settings. For instance, soaking *Drosophila melanogaster* larvae in dsRNA solution targeting glucuronidase successfully suppresses enzymatic activity by 50% and confirms effective dsRNA uptake via intestinal tracts [31,76].

**Figure 3:**
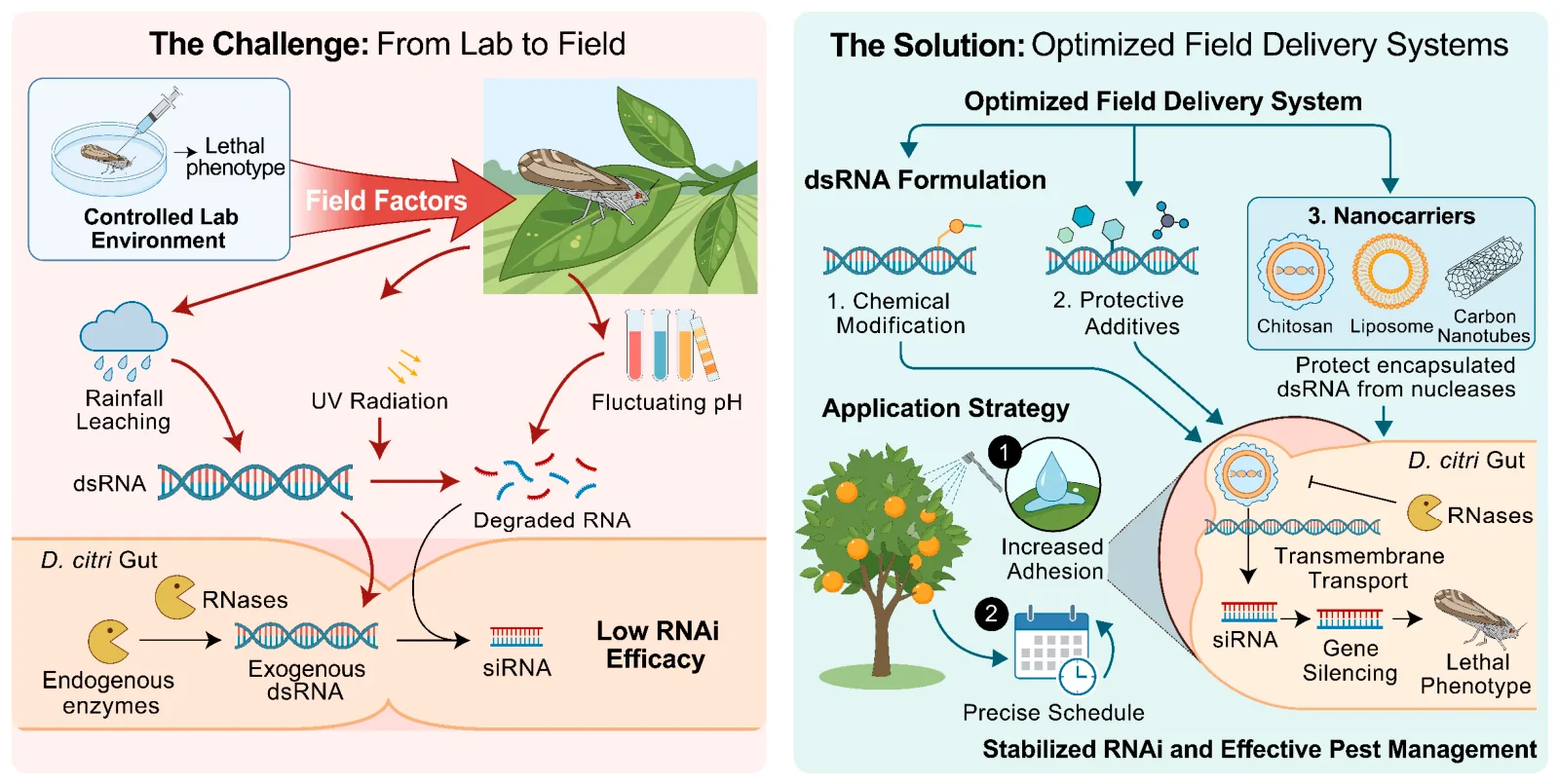
Schematic diagram of obstacles hampering the translation of laboratory RNAi effects to field conditions and optimized field delivery strategies for *D. citri*. Field environmental degradation and intestinal RNase digestion jointly break down dsRNA and reduce RNAi efficiency. To solve this problem, dsRNA can be stabilized via chemical modification, protective additives and nanocarrier packaging, combined with optimized spraying adhesion and timed application strategies, so as to stabilize RNAi efficiency and realize field psyllid control.

Intrinsic biological barriers within *D. citri* further weaken field RNAi effects. Endogenous RNases in the psyllid digestive system rapidly degrade exogenous dsRNA, shorten its effective duration, attenuate gene silencing and make lethal phenotypic changes difficult to observe [77]. Similar degradation patterns also cause low RNAi efficiency in Lepidoptera insects, where insufficient intracellular processing of dsRNA into siRNA restricts silencing performance [78,79]. A previous review has summarized the variations in RNAi performance across different insect orders: Lepidoptera exhibit low RNAi efficiency, Coleoptera show robust environmental RNAi, and Orthoptera possess efficient systemic RNAi. It highlights that divergent dsRNase activity constitutes one of the key factors responsible for inter-order differences in RNAi sensitivity [80]. Accordingly, core optimization directions for field delivery systems focus on enhancing dsRNA stability against environmental and enzymatic degradation. Chemical modification of dsRNA and protective additives are conventional strategies to resist degradation, while nanocarriers such as chitosan, liposomes and carbon nanotubes have emerged as promising delivery materials to encapsulate dsRNA, prevent nuclease cleavage and facilitate transmembrane transport across psyllid gut barriers [34,81,82,83,84]. Formulation optimization is also essential: surfactants and auxiliary agents improve the adhesion of dsRNA sprays on citrus leaf surfaces and increase ingestion probability by psyllids. Meanwhile, application timing and dosage should be matched to the feeding rhythm and developmental cycle of *D. citri*, maximizing silencing efficiency with minimal dsRNA consumption (Figure 3).

Overall, the transformation of RNAi technology from laboratory research to field prevention essentially requires breaking dual barriers from environmental degradation and insect endogenous degradation. Optimized delivery formulations, biocompatible nanocarriers and precise application schedules jointly improve environmental adaptability of dsRNA preparations. Rational configuration of field delivery systems not only stabilizes RNAi control effects in orchards, but also lays the groundwork for developing spray-type RNA biopesticides targeting citrus psyllids.

## 4. Conclusions and Future Perspectives

Great achievements have been made in RNAi-based control against *D*. *citri*, yet multiple bottlenecks still hinder the translational application of this technology from laboratory trials to citrus orchards. Future research should center on four major orientations: deeper excavation of target genes, improvement in dsRNA delivery efficiency and stability, comprehensive ecological safety assessment, and synergistic integration with alternative green pest management tactics. For target-gene exploration, researchers should combine high-throughput RNAi screening with available whole-genome datasets of *D. citri* to systematically mine functionally essential genes with tissue-specific or developmental-stage-specific expression patterns under distinct physiological conditions [85]. Given that single-gene RNAi treatment readily induces pest resistance, rational design of multi-target dsRNA constructs that simultaneously silence multiple non-redundant vital genes is proposed as a superior alternative to improve control efficacy and delay resistance evolution. For dsRNA delivery innovation, research should prioritize the screening and development of low-cost, biocompatible nanomaterials. These nanocarriers are expected to shield dsRNA against environmental degradation and facilitate its absorption and intracellular transport inside psyllids. Quantitative experimental characterization is urgently required to disentangle the interactions between dsRNA-nanomaterial complexes and psyllid gut epithelium, together with systematic evaluation of nanocarrier biosafety, persistent efficacy, leaf-surface adhesion and plant translocation characteristics under orchard-mimicking conditions [86]. Meanwhile, transgenic citrus plants capable of constitutively producing psyllid-specific dsRNA can be created via transgenesis or advanced gene-editing techniques. To guarantee the species specificity of RNAi agents and avoid adverse impacts on non-target organisms, bioinformatic homology alignment between dsRNA sequences and genomes of non-target species, together with feeding bioassays, should be performed for systematic safety evaluation. To guarantee the species specificity of RNAi agents and avoid adverse impacts on non-target organisms, a tiered safety-assessment framework combining in silico prediction and laboratory biological verification should be implemented. For in silico evaluation, high-throughput homology searches against genome datasets of local beneficial arthropods, pollinators and natural enemies are required to eliminate dsRNA candidates carrying conserved short-sequence motifs that may trigger off-target silencing. Subsequently, laboratory-level feeding bioassays using representative non-target species are indispensable to validate real-world biological risks, because purely computational prediction cannot fully exclude unpredicted off-target events. Therefore, bioinformatic homology alignment between dsRNA sequences and genomes of non-target species, together with feeding bioassays, should be performed to establish standardized workflows for systematic safety evaluation, which is also a core technical requirement for the regulatory registration of RNAi-based biopesticides.

At present, the molecular mechanisms underlying RNAi resistance in *D. citri* remain poorly understood. Key resistance pathways including target-site mutation and upregulated expression of dsRNA-degrading nucleases have not been thoroughly characterized. Previous studies on model organisms such as *Drosophila melanogaster* have uncovered diverse molecular pathways enabling pests to evade RNAi suppression. For example, the RESC rescue protein system can prevent complete silencing of target sequences, which is applicable not only to fruit flies but also to other organisms, providing novel tools for gene functional research and pest management [87,88]. To fill this research gap, future work should carry out forward genetic screening and transcriptomic profiling of resistant psyllid populations to identify core resistance-related molecular modules. Additionally, insufficient attention has been paid to the evolutionary risk of RNAi resistance, and mature resistance management strategies are still lacking. Proactive resistance mitigation frameworks should be established in advance by drawing lessons from *Bt*-crop resistance-management experiences [89,90]. Concrete tactics include rotational application or combined usage of dsRNA molecules with distinct modes of action, as well as integration of dsRNA agents with biopesticides and low-toxicity agrochemicals to effectively delay the emergence and spread of resistance [13,91]. RNAi technology is not meant to be applied independently; instead, it serves as a core component within the integrated management system of citrus HLB disease. Follow-up research needs to explore the compatibility and synergistic effects between low-dose highly specific dsRNA and natural enemies such as parasitoid wasps. Combination formulations of dsRNA with insect pheromones or repellents can interfere with psyllid behaviors while causing lethal effects via ingestion. Applying RNAi biopesticides at the early outbreak stage of psyllid populations enables precise population suppression, cuts the usage of broad-spectrum chemical insecticides, and eventually facilitates environmentally friendly and sustainable citrus cultivation. In general, resolving delivery stability, biosafety and resistance risks will promote the large-scale popularization of RNAi technology and realize green prevention and control of citrus HLB transmitted by *D. citri*.

## Data Availability

The original contributions presented in this study are included in the article. Further inquiries can be directed to the corresponding author.

## Abbreviations

The following abbreviations are used in this manuscript:

HLB	Huanglongbing
RNAi	RNA interference
*C*Las	*Candidatus* Liberibacter asiaticus
dsRNA	double-stranded RNA
